# Supplementary material for: Single-unit activations confer inductive biases for emergent circuit solutions to cognitive tasks
Source: Nat Mach Intell. 2025 Oct 20;7(10):1742–54. doi: 10.1038/s42256-025-01127-2 (PMC12552134; doi:10.1038/s42256-025-01127-2)
Supplement: Supplementary file 1 — Reporting Summary [file 42256_2025_1127_MOESM1_ESM.pdf]

nature portfolio

Corresponding author(s): Tatiana Engel

Last updated by author(s): Aug 9, 2025

Reporting Summary

Nature Portfolio wishes to improve the reproducibility of the work that we publish. This form provides structure for consistency and transparency in reporting. For further information on Nature Portfolio policies, see our Editorial Policies and the Editorial Policy Checklist.

Statistics

For all statistical analyses, confirm that the following items are present in the figure legend, table legend, main text, or Methods section.

n/a	Confirmed	
☐	☒	The exact sample size (*n*) for each experimental group/condition, given as a discrete number and unit of measurement
☐	☒	A statement on whether measurements were taken from distinct samples or whether the same sample was measured repeatedly
☒	☐	The statistical test(s) used AND whether they are one- or two-sided<br>*Only common tests should be described solely by name; describe more complex techniques in the Methods section.*
☒	☐	A description of all covariates tested
☒	☐	A description of any assumptions or corrections, such as tests of normality and adjustment for multiple comparisons
☐	☒	A full description of the statistical parameters including central tendency (e.g. means) or other basic estimates (e.g. regression coefficient) AND variation (e.g. standard deviation) or associated estimates of uncertainty (e.g. confidence intervals)
☒	☐	For null hypothesis testing, the test statistic (e.g. *F*, *t*, *r*) with confidence intervals, effect sizes, degrees of freedom and *P* value noted<br>*Give P values as exact values whenever suitable.*
☒	☐	For Bayesian analysis, information on the choice of priors and Markov chain Monte Carlo settings
☒	☐	For hierarchical and complex designs, identification of the appropriate level for tests and full reporting of outcomes
☒	☐	Estimates of effect sizes (e.g. Cohen's *d*, Pearson's *r*), indicating how they were calculated

Our web collection on statistics for biologists contains articles on many of the points above.

Software and code

Policy information about availability of computer code

Data collection	Synthetic data were generated using custom Python 3 code. The source code to reproduce results of this study is freely available on GitHub: https://github.com/engellab/trainRNNbrain , https://github.com/engellab/latent_circuit_inference , https://github.com/engellab/ActivationMattersRNN . The analyzed synthetic data are available at https://github.com/engellab/ActivationMattersRNN/tree/main/data
Data analysis	Data analysis was performed using custom Python 3 code available at https://github.com/engellab/ActivationMattersRNN.

For manuscripts utilizing custom algorithms or software that are central to the research but not yet described in published literature, software must be made available to editors and reviewers. We strongly encourage code deposition in a community repository (e.g. GitHub). See the Nature Portfolio guidelines for submitting code & software for further information.

Data

Policy information about availability of data

All manuscripts must include a data availability statement. This statement should provide the following information, where applicable:

- Accession codes, unique identifiers, or web links for publicly available datasets
- A description of any restrictions on data availability
- For clinical datasets or third party data, please ensure that the statement adheres to our policy

All synthetic data reported in this paper can be reproduced using the source code. The data are also available at https://github.com/engellab/ActivationMattersRNN/tree/main/data.

Human research participants

Policy information about studies involving human research participants and Sex and Gender in Research.

Reporting on sex and gender	NA
Population characteristics	NA
Recruitment	NA
Ethics oversight	NA

Note that full information on the approval of the study protocol must also be provided in the manuscript.

Field-specific reporting

Please select the one below that is the best fit for your research. If you are not sure, read the appropriate sections before making your selection.

☒ Life sciences ☐ Behavioural & social sciences ☐ Ecological, evolutionary & environmental sciences

For a reference copy of the document with all sections, see nature.com/documents/nr-reporting-summary-flat.pdf

Life sciences study design

All studies must disclose on these points even when the disclosure is negative.

Sample size	We trained 100 RNNs with each architecture ({Dale, No Dale} x {ReLU, tanh, sigmoid}) on each task, providing a robust sample size for our analyses. No statistical methods were used to predetermine sample size. The sample size used here substantially exceeds that of similar previous studies; for example, Maheswaranathan et al. (NeurIPS 2019) used 30 RNNs per architecture.
Data exclusions	No data were excluded
Replication	We trained 100 RNNs with each architecture on each task. The results were replicated between the top 50 RNNs with the best task performance and the remaining 50 RNNs.
Randomization	Randomization is not relevant to this study. RNNs were not allocated into groups. All RNNs were analyzed using the same processing functions and procedures.
Blinding	Blinding is not relevant to this study. RNNs were not allocated into groups. All RNNs were analyzed using the same processing functions and procedures.

Reporting for specific materials, systems and methods

We require information from authors about some types of materials, experimental systems and methods used in many studies. Here, indicate whether each material, system or method listed is relevant to your study. If you are not sure if a list item applies to your research, read the appropriate section before selecting a response.

Materials & experimental systems

n/a	Involved in the study	
☒	☐	Antibodies
☒	☐	Eukaryotic cell lines
☒	☐	Palaeontology and archaeology
☒	☐	Animals and other organisms
☒	☐	Clinical data
☒	☐	Dual use research of concern

Methods

n/a	Involved in the study	
☒	☐	ChIP-seq
☒	☐	Flow cytometry
☒	☐	MRI-based neuroimaging
